# Psychological Impact of the COVID-19 Pandemic on Children and Adolescents With and Without Mental Disorders

**DOI:** 10.3389/fpubh.2021.679041

**Published:** 2021-11-05

**Authors:** Susanne Gilsbach, Beate Herpertz-Dahlmann, Kerstin Konrad

**Affiliations:** ^1^Department of Child and Adolescent Psychiatry, Psychosomatics and Psychotherapy, Medical Faculty, RWTH Aachen University, Aachen, Germany; ^2^Child Neuropsychology Section, Department of Child and Adolescent Psychiatry, Psychosomatics and Psychotherapy, Medical Faculty, RWTH Aachen University, Aachen, Germany; ^3^JARA-Brain Institute II, Molecular Neuroscience and Neuroimaging, RWTH Aachen & Research Centre Juelich, Juelich, Germany

**Keywords:** COVID-19 pandemic, mental health children and adolescents, mental disorder, psychological burden, quarantine

## Abstract

**Background:** The previous and current studies highlight the psychological distress caused by coronavirus disease 2019 (COVID-19)-associated restrictions among the general population, especially among children and adolescents; however, few studies have examined children and adolescents with a mental disorder. The current study aimed to explore whether youth with mental disorders show a higher pandemic-associated psychological burden than healthy children and adolescents and to determine which psychiatric diagnoses are particularly associated with a higher distress level.

**Methods:** In this study, 144 children and adolescents between the ages of 6 and 18 years with a mental disorder and 48 children and adolescents within the same age range without a mental disorder, and their caregivers, completed questionnaires assessing the pandemic-associated trauma symptoms (the Child Report of Post-Traumatic Symptoms [CROPS] and the Parents Report of Post-Traumatic Symptoms [PROPS]). Additionally, we asked specific questions about the pandemic-associated stress factors, such as financial problems, prolonged screen times, or loneliness.

**Results:** Children and adolescents with a mental illness showed a significantly higher psychological burden than their mentally healthy peers. Female gender was a risk factor for a higher self-reported psychological burden, and younger age was associated with a more extensive parent-reported psychological burden. The patients with a depressive disorder showed significantly higher levels of psychological distress associated with the COVID-19 pandemic than the patients with an attention deficit and/or a conduct disorder.

**Conclusions:** Children and adolescents with a mental illness, particularly, female children and individuals with a depressive disorder, are at an increased risk of suffering from pandemic-associated psychological distress. Adequate mental health care options, such as telepsychiatry, are indispensable.

## Introduction

The coronavirus disease 2019 (COVID-19) pandemic is an unprecedented event, and the extent and severity of the pandemic have affected the whole world. The daily life of almost everyone has changed dramatically, and the future of the pandemic remains unpredictable. Many countries have implemented strong restrictions concerning social contacts and mobility. The schools and public institutions have been closed repeatedly, and many families have been quarantined for more than a week. A widespread climate of fear and uncertainty adds to the burden.

The previous studies have highlighted the negative effects of quarantine or natural disasters on the mental and physical well-being of children and adolescents. In particular, reduced physical activity, weight gain, a decrease in cardiorespiratory fitness, prolonged screen times, irregular sleep patterns, and less appropriate diets have been described [refer to (1), for a review). Sprang and Silman (2) found that the posttraumatic stress scores in children who had been quarantined for various infectious diseases were four times higher than the scores of children who were not quarantined. In the studies conducted during epidemics and pandemics, the adults have reported fears of infection, frustration, boredom, financial loss, and lack of interpersonal contacts (3).

During the COVID-19 pandemic, several studies showed higher rates of psychological distress, anxiety, depression, and posttraumatic stress in the general population across many countries. For example, Biondi et al. (4) investigated a large sample of undergraduate students and found that the immature defense mechanisms and internalizing personality traits increased the risk for depression, anxiety, and stress symptoms, which were in turn associated with the lower levels of compliance with national healthcare measures. A survey of the general Italian population during the COVID-19 pandemic revealed the female gender to be associated with the higher levels of affective symptoms and having an acquaintance infected was associated with depression and stress (5). In a recent review, the risk factors associated with mental distress included female gender, younger age group (<40 years), presence of chronic or mental illness, unemployment, student status, and frequent exposure to social media and news concerning COVID-19 [refer to (6)].

Recently, a growing amount of research has been dedicated to the effects of the current pandemic on children and adolescents (7).

In an online survey during the COVID-19 pandemic, the Chinese adolescents between ages 12 and 18 showed rates of depressive and anxiety symptoms as high as 43.7 and 37.4%, respectively, with the female gender being a risk factor for a higher symptom burden (8). Duan et al. (9) accordingly found that being female as well as having family members or friends infected with coronavirus increased the pandemic-associated anxiety among children and adolescents. In a recent epidemiological study in Germany, two-thirds of the children and adolescents reported being highly burdened by the COVID-19 pandemic. They experienced significantly lower quality of life, and the mental health problems almost doubled with the higher anxiety levels than before the pandemic. The children with low socioeconomic status, migration background and limited living space were significantly more affected (10).

Lee (11) summarized the mental health effects of school closures during COVID-19 and particularly, stressed the effects on children and adolescents with mental health needs, referring to a survey by the mental health charity “YoungMinds,” that included the participants up to 25 years of age with a mental illness history in the United Kingdom. A total of 83% of respondents reported a worsening of their conditions, and 26% indicated being unable to access health support. The peer support groups and face-to-face services were canceled, and access to online support seemed to be challenging for many children and adolescents (https://youngminds.org.uk). Lee also emphasized the importance of schools as an anchor in the life of children with mental health needs and the necessity of further research to monitor the effects of school closures, distancing measures, and the pandemic on the well-being of children and adolescents, especially those with a mental disorder. As shown by Reiss et al. (12) before the pandemic, stressful life events and a low socioeconomic status generally increase the risk of reporting mental health problems in children and adolescents aged between 7 and 17 years. However, the mental health problems at baseline were the best predictor for mental health problems in a 2 year follow-up.

The current study aimed to explore whether youth with mental disorders show a higher pandemic-associated psychological burden than healthy children and adolescents and which psychiatric diagnoses are particularly associated with a higher distress level. We hypothesized that the children and adolescents in psychiatric care are more distressed by the COVID-19 pandemic than the children recruited from the community without mental health problems and that internalizing disorders, such as depression, trauma, obsessive-compulsive disorder (OCD), and anxiety, as well as female gender are the risk factors for more detrimental psychological consequences.

## Materials and Methods

### Participants

During the first lock-down of the pandemic crisis in Germany in spring 2020, we recruited 147 patients (48 inpatients, 99 outpatients) aged 6–18 years who received treatment at the Department of Child and Adolescent Psychiatry, University Hospital in Aachen, Germany. The patients were included irrespective of the type of their diagnosis.

Additionally, we recruited 48 healthy controls (HCs) from 6 to 18 years of age from the community who had participated in the former studies and consented to be recontacted.

The samples did not differ significantly regarding age or type of school. However, the gender distribution differed significantly, with more female gender in the patient group and more male gender in the HC group.

The sample characteristics are shown in Table 1.

**Table 1:** Sample characteristics.

	**Patients** ***n*** **= 147**	**Outpatients** ***n*** **= 99**	**Inpatients** ***n*** **= 48**	**Healthy controls** ***n*** **= 48**	***p***
**Age**					
Mean (SD) (years)	13.3 (3.0)	13.3 (2.9)	13.3 (3.1)	13.5 (3.0)	n.s.
**Gender**					0.005
Female (%)	55.0	51.5	64.9	33.3	
Male (%)	43.6	46.9	35.1	66.7	
Missing (%)	0.7	1	0	0	
**School**					n.s.
Elementary school (%)	16.1	15.2	18	16.7	
Lower secondary school (%)	12.8	14.1	10	0	
Middle secondary school (%)	6	4	10	4.2	
Higher secondary school (%)	60.4	61.1	58	79.2	
University studies	0.7	1	0	0	
Missing (%)	4	4	4		
	*n* = 126			*n* = 48	
**CROPS** total mean (SD)	14.14 (10.1)		5.38 (4.9)	<0.001	
	*n* = 107			*n* = 44	
**PROPS** total mean (SD)	14.7 (19.7)			5.3 (4.5)	<0.001

*SD, standard deviation. Results from ANOVA/T-tests for group differences*.

Depressive (24.9%) and attention deficit/hyperactivity disorder (ADHD) with and without conduct disorder (38.3%) were the most frequent diagnoses. A total of 4.1% of the patients had a trauma diagnosis prior to the pandemic. Parental informed consent and assent of the children were obtained, and the study was approved by the local ethical committee (EK 218/20) and conducted in accordance with the Code of Ethics of the World Medical Association (Declaration of Helsinki).

### Materials

We used the German version of the Child Report of Post-Traumatic Symptoms (CROPS) and the Parents Report of Post-Traumatic Symptoms (PROPS) to assess the COVID-19-associated distress. The CROPS, originally developed, is a self-report measure for children and adolescents that assesses a broad range of posttraumatic symptoms, with or without an identified trauma, and can be used to measure changes in symptomatology over time (13). The parent version of the PROPS, also developed, does not contain identical items as the CROPS; however, the items are derived from the same item pool. The CROPS focuses on the thoughts and feelings (internal processes), whereas the PROPS focuses on the observable behaviors (externals), with cut-off scores of 19 and 16, respectively (13). In the instructions, we were asked specifically to answer the questions with respect to the pandemic-associated consequences.

In addition, we asked questions (self and parent report) regarding more general effects of the COVID-19 pandemic and quarantine [as derived from (3)] concerning the financial consequences, changes in bullying experiences, loneliness, sleeping patterns, eating habits and physical activity, screen time, and experiences of loss or burden by infected or deceased family members or friends. The questions were answered on a 5-point Likert scale from −2 not at all to 2 very much.

The internal consistencies for the original versions of the CROPS/PROPS questionnaires were described as ranging between 0.8 and 0.92 for the self-report (https://www.nctsn.org/measures/child-report-post-traumatic-symptoms) and between 0.87 and 0.9 for the parental report (https://www.nctsn.org/measures/parent-report-post-traumatic-stress-symptoms). In the current sample (including all the participants), Cronbach's alpha was 0.88 for the CROPS plus added questions and 0.88 for the PROPS plus added questions.

### Statistical Analysis

All the statistical analyses were conducted using IBM SPSS, version 27, Armonk, NY with the AMOS module. We compared total CROPS as well as PROPS scores using two-sided Student's *t*-tests to examine the differences between the patient group and HCs. We also calculated the number/percentage of participants scoring above the cut-off in the questionnaire and compared them between the groups by means of chi-square tests. To determine the associations between self and parental reports, Pearson's correlation coefficients were calculated between CROPS and PROPS scores, separately within the patient and the HC group. Between-group differences between the pandemic-associated psychological burden in patients and HCs were further controlled for the effects of gender, age, and school type based on the findings by Ravens-Sieberer et al. (10) and Reiss et al. (12) by means of covariance analyses. The same covariance analysis was conducted for the answers to our pandemic-specific added questions (again separately for the self and parent reports), using the mean total scores of the individual answers as dependent and patient status, gender, and age as independent variables.

To explore the risk factors associated with particularly elevated pandemic-associated psychological burden in the children and adolescents with pre-existing mental disorders, we conducted multiple linear regression analyses to predict the total CROPS and PROPS scores (as dependent variables) within the patient group only. Based on our *a-priori* hypothesis that internalizing mental disorders (i.e., depression, anxiety, and trauma-related disorders) were associated with more pandemic-associated burden than the externalizing disorders (i.e., ADHD and conduct disorder) and that the trauma scores varied depending on the age of a subject (10), gender (6, 8), experienced closeness to the pandemic (6, 9), as well as type of school (as a tangible measure of the education level of the child and alternative indicator for socioeconomic status in adolescents) as representative for socioeconomic status (14, 15). We entered the type of diagnosis, gender, age, the family member with a COVID-19, and type of school as predictors as well as all the possible two- and three-way interactions between these predictors in the multiple regression analyses. These predictors were selected *a-priori* based on the previous findings in the non-clinical samples [refer to (6), for a review]. To reduce the risk of sample bias, we only included mental disorders with more than 10 patients each as predictors in the analyses: eating disorders (*n* = 11), depressive disorders (*n* = 20), anxiety disorders, OCD, and posttraumatic stress disorder (PTSD) (*n* = 29), and ADHD and conduct disorder (*n* = 42). The dummy variables were used for coding the mental disorders. The number of predictors was limited to seven to avoid overfitting (16). We calculated a *post-hoc* power analysis with the program G⋆power3.1.9.6 and found that the statistical power achieved for a sample size of 102 subjects, an effect size of f^2^ = 0.15, an alpha level of 0.05, and seven predictors was 0.8 which was considered sufficient.

## Results

The patients and HCs differed significantly regarding their pandemic-associated psychological burden according to both, self (CROPS) and parental report (PROPS) (as shown in Table 1).

A total of 24.8% of the patients (*n* = 132) and none of the HCs (*n* = 48) reached the CROPS cut-off for clinically significant pandemic-associated distress (chi^2^ = 16.94, *p* = < 0.001). A total of 34.2% of the patients (*n* = 113) and 4.2% of the controls (*n* = 44) reached the PROPS cut-off (as rated by their parents) for clinically significant pandemic-associated distress (chi^2^ = 23.33, *p* = < 0.001).

The total scores on the CROPS and PROPS were significantly associated with each other in both the children with (*r* = 0.51, *p* < 0.001) and without (*r* = 0.32, *p* = 0.03) mental disorders.

The covariance analyses revealed a significant main effect for patient status and gender with respect to the CROPS scores with no significant interaction between these factors. The female gender and patients with a mental disorder had higher CROPS scores than the male gender and HCs, respectively. For the parent-rated PROPS scores, significant main effects were found for patient status and age and no interaction effects. The patients with a mental illness reached higher PROPS scores, and the PROPS scores were negatively associated with age. All other factors were not significant (as shown in Table 2).

**Table 2:** ANCOVA with CROPS/PROPS total scores as dependent and age, patient status, gender, and type of school as independent variables.

	**df**	**Mean of squares**	**F**	***P***
**Dependent variable: CROPS scores**				
Age	1	31.91	0.45	0.50
Patient status	1	586.26	8.28	0.005^*^
Gender	1	413.23	5.84	0.017^*^
Type of school	3	82.47	1.17	0.33
Patient status × gender	1	11.32	0.16	0.69
Patient status × type of school	2	94.98	1.34	0.26
Gender × type of school	3	181.90	2.57	0.06
Patient status × gender × type of school	1	10.99	0.155	0.69
**Dependent variable: PROPS scores**				
Age	1	371.47	4.37	0.038^*^
Patient status	1	1037.40	12.21	0.001^**^
Gender	1	195.43	2.30	0.13
Type of school	3	113.16	1.33	0.27
Patient status × Gender	1	3.78	0.04	0.83
Patient status × type of school	2	47.60	0.56	0.57
Gender × type of school	3	126.98	1.49	0.22
Patient status × gender × type of school	1	4.34	0.05	0.82

* *p < 0.05*,

** *p < 0.001*.

Regarding the pandemic-specific questions, a multivariate analysis of covariance controlling for gender and age showed no significant difference between the patients and HCs in the self-reported amount of boredom [*F*_(1,137)_ = 0.19, *p* = 0.66], loneliness [*F*_(1,137)_ = 0.25, *p* = 0.62], physical activity [*F*_(1,137)_ = 0.11, *p* = 0.74], eating habits [*F*_(1,137)_ = 0.75, *p* = 0.39], screen time [*F*_(1,137)_ = 0.94, *p* = 0.34], fear of infection of family members [*F*_(1,137)_ = 2.72, *p* = 0.09], or fear of infecting others [*F*_(1,137)_ = 0.68, *p* = 0.41]. Accordingly, the parent report scores did not differ on these items (boredom [*F*_(1,116)_ = 1.21, *p* = 0.27], loneliness [*F*_(1,116)_ = 1.71, *p* = 0.19], physical activity [*F*_(1,116)_ = 2.19, *p* = 0.14], eating habits [*F*_(1,116)_ = 1.8, *p* = 0.18], screen time [*F*_(1,116)_ = 3.58, *p* = 0.06], fear of infection of family members [*F*_(1,116)_ = 3.56, *p* = 0.07], or fear of infecting others [*F*_(1,116)_ = 0.9, *p* = 0.35]).

However, the self-reported amount of experienced bullying was described as significantly more reduced during quarantine in the patients compared with HCs [*F*_(1,137)_ = 14.22, *p* < 0.001]. Furthermore, the patients reported a greater increase in sleep disturbances during the pandemic crisis [*F*_(1,137)_ = 9.22, *p* = 0.003]. Finally, both the groups reported fewer financial problems before the COVID-19 pandemic, which were even more so in the patient group [*F*_(1,137)_ = 6.96, *p* = 0.009].

In the parent reports, the patients and HCs differed only regarding the experienced bullying, confirming the higher self-reported decrease in patients [*F*_(1,116)_ = 6.38, *p* = 0.01].

**Graph 1:**
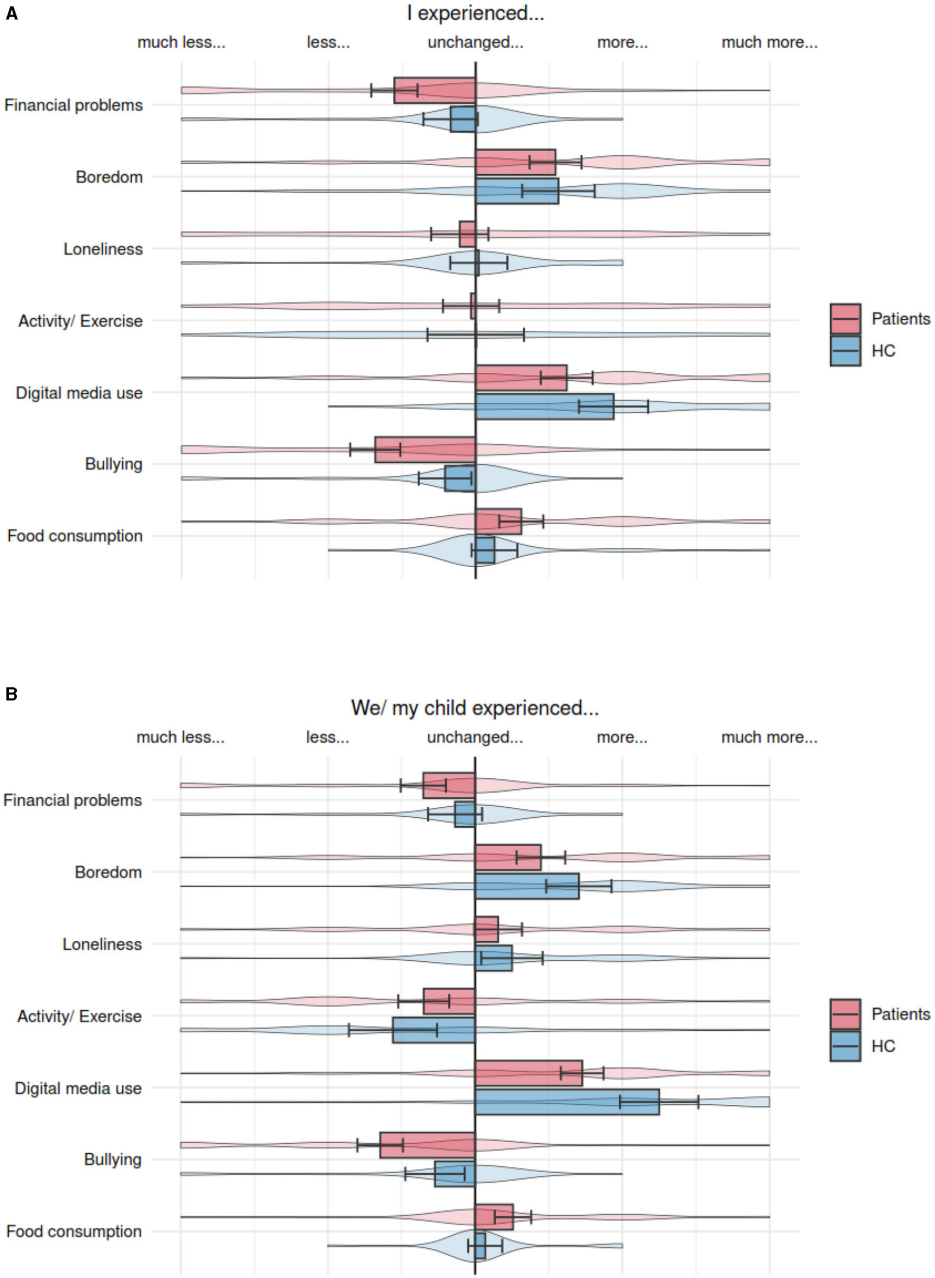
**(A)** Self-reported answers to pandemic-specific questions. **(B)** Parent-reported answers to pandemic-specific questions.

As shown in Graphs 1A,B. Graph 1A: Self-reported answers to pandemic-specific questions. Graph 1B: Parent-reported answers to pandemic-specific questions.

**Graph 2:**
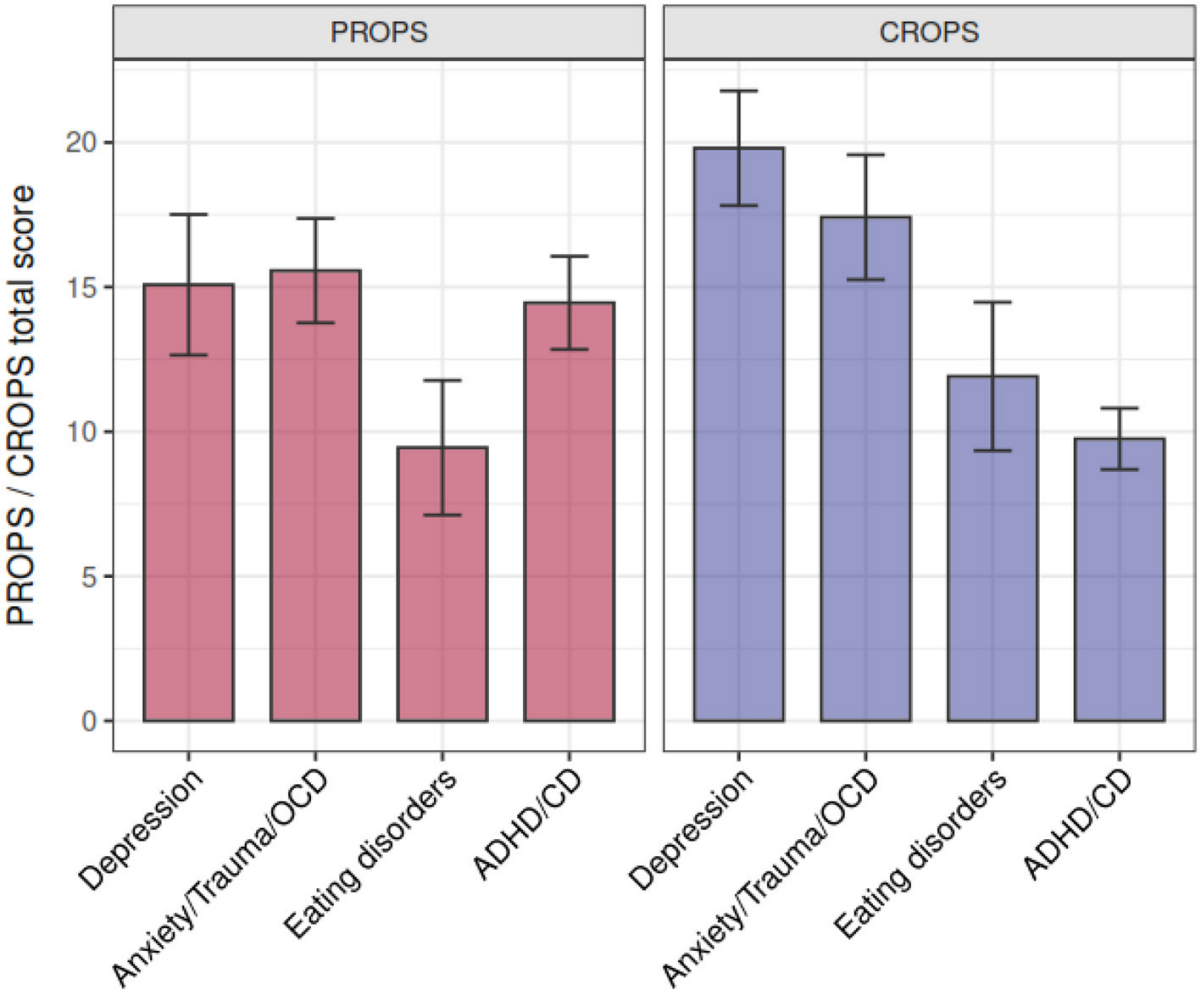
Parents Report of Post-Traumatic Symptoms (PROPS) and Child Report of Post-Traumatic Symptoms (CROPS) scores depending on the clinical diagnosis. CD, conduct disorder.

The psychological burden separately for diagnostic groups of mental disorders is depicted in Graph 2.

As shown in Graph 2: Parents Report of Post-Traumatic Symptoms (PROPS) and Child Report of Post-Traumatic Symptoms (CROPS) scores depending on the clinical diagnosis.

The multiple regression analyses of risk factors revealed that the CROPS scores were significantly predicted by the model, which explained 26.6% of the variance [*F*_(6,93)_ = 5.63, *p* = 0.001]. Gender was a significant predictor for the higher CROPS scores, while age and a family member with a COVID-19 infection were not significant predictors. Having a depressive disorder predicted higher CROPS scores. The remaining diagnostic groups did not explain a significant amount of variance.

The model explained 18.2% of the variance in PROPS scores [*F*_(7,78)_ = 2.48, *p* = 0.024]. Age was a significant predictor for pandemic-associated distress, while gender or type of diagnosis was not.

Table 3 shows a regression for CROPS and PROPS scores, predictors: Age and gender, type of diagnosis as dummy variable: Depressive disorders, anxiety/OCD/trauma, eating disorders with externalizing disorder as an excluded variable, and family member or friend with COVID-19.

**Table 3:** Regression for CROPS and PROPS scores, predictors: age and gender, type of diagnosis as dummy variable: depressive disorders, anxiety/OCD/trauma, eating disorders with externalizing disorder as excluded variable, and family member or friend with Covid-19.

	**Standardized beta**	**T**	***P***
**Dependent variable: CROPS scores**			
Age	−0.035	−0.36	0.72
Gender	−0.32	−2.77	0.007^*^
Depressive disorder	0.26	2.13	0.036^*^
Anxiety/OCD/trauma	0.19	1.52	0.13
Eating disorder	−0.07	−0.62	0.54
Family member or friend with Covid-19	−0.14	−1.61	0.11
**Dependent variable: PROPS scores**			
Age	−0.36	−3.26	0.002
Gender	−0.03	−0.22	0.83
Depressive disorder	0.12	0.91	0.37
Anxiety/OCD/trauma	0.13	0.98	0.33
Eating disorder	−0.06	−0.46	0.65
Family member or friend with Covid-19	−0.19	−1.80	0.08

* *p < 0.05*.

## Discussion

In this study, the COVID-19 pandemic-associated psychological burden was assessed in the patients with mental disorders in clinical care compared with HCs by using self- and parent-reported questionnaires.

We found a striking difference in self- and parent-reported psychological distress between mentally ill children and adolescents and HCs. This finding must be interpreted in the light of various aspects. Children and adolescents with a mental disorder are more vulnerable and less resilient than healthy peers, thus they might suffer more from stressful life circumstances. Furthermore, the pandemic-induced restrictions impaired access to mental healthcare substantially (https://youngminds.org.uk). The “novelty” of this crisis in Germany, in particular during the first lock-down, meant a lack of viable alternatives to face-to-face treatment, such as telepsychiatry [e.g., (17)]. Finally, although we asked specifically for pandemic-associated symptoms in the instruction of the questionnaires, the higher trauma scores of the patient group might reflect a mixture of a trauma-related history associated with the development of general psychopathology [e.g., (18)] and the specific emotional burden due to the pandemic.

Female gender was the main factor that influenced the severity of the self-reported pandemic-associated psychological burden in our entire sample, such as the participants with and without a pre-existing mental disorder. This gender effect on the self-reported stress of the pandemic crisis has been previously described for the general population as described by Wang et al. (1) in the adults as well as by Duan et al. (9) for children. Of note, there was no significant interaction effect between “gender” and “mental disorders” on the psychological burden, thus, both the factors seem to independently contribute to the more pandemic-associated experience of stress. In addition, Ravens-Sieberer et al. (10) also reported in a German population-wide study that children of younger age are at particular risk for higher psychological burden. This corresponds well to our finding of an increased parent-reported psychological burden in younger children. This finding may be due to younger children being less cognitive and able to understand the changes of the quarantine situation. Alternatively, the younger children might also suffer more from social distancing. Finally, the parents might be more prone to acknowledge psychological distress in younger children.

There was a decrease in experienced bullying in all the participants, but this decrease was significantly higher in the patient group. This finding is plausible since the lack of social contacts leads to reduced opportunities to be bullied by peers. The higher decrease in patients might have been caused by the fact that the children and adolescents with psychological problems experience more bullying than their mentally healthy peers (19). It must be noted, however, that the question did not differentiate between cyberbullying and in-person bullying. Finally, we found that all the participants ate more and showed less physical activity (although only small effects) in both the groups, in line with the previous studies (1, 11).

The risk analysis in the patient group supported our hypothesis that among the mentally ill patients, depressive disorders increase the risk factors for an increased pandemic-induced psychological burden. This effect was only found in the self-but not in the parent reports indicating that the parents might not be aware of the pandemic-specific burden of this patient group. Thus, it might be very important to ask the children and adolescents with depressive disorders themselves about their emotions and worries during crisis situations. Contrary to our hypothesis, other internalizing disorders, such as anxiety disorders or stress-related disorders, such as PTSD, were not significant risk factors for increased psychological burden. Note, however, that this might be due to a lack of power as this patient group showed the second-highest self-reported pandemic-associated psychological burden (as shown in Graph 2). Furthermore, also in our multiple regression analysis conducted in patients only, the female gender was a significant risk factor for higher self-reported burden, again with no interactions with other risk factors. However, it must be kept in mind that adolescent girls, as well as adult female girls, are in general more prone to depressive disorders than their male peers (1, 20) which might have biased this finding. For the parent reports in the patient group, our multiple regression analyses could not confirm any specific diagnostic group to be specifically associated with higher psychological distress, but only younger age was revealed as a significant predictor for emotional distress due to the pandemic. Thus, the parental ratings might be biased by considerations of developmental stage/cognitive immaturity of their children and might overlook the emotional burden of highly vulnerable adolescents, i.e., with pre-existing depressive disorders.

### Limitations

The current findings, however, should be considered in the context of some limitations. The current study only included one survey timepoint and pre-pandemic and follow-up data are lacking. The recent studies hint at rather a volatile psychopathology over the timespan of the pandemic with an increase of psychological distress, in the beginning, a certain remission during the summer of 2020, and a subsequent deterioration (21). Also, psychopathology due to pre-existing morbidity cannot be differentiated completely from pandemic-induced distress. Thus, our findings need to be replicated in larger samples, and with longitudinal follow-up assessments.

## Conclusions and Future Directions

Our results point to the quarantine-associated mental health risks, such as symptoms of depression, low mood, irritability, insomnia, anger, emotional exhaustion, and PTSD in children and adolescents. As Fegert et al. (7) criticize, data on these detrimental effects on the mental health of children are scarce, and as Nearchou et al. (22) point out, research quality in this domain is low. The current study confirms the risk for a substantial psychological burden and highlights the importance of further research in this area. As a high-risk group for maladaptive psychological functioning, the children and adolescents with a mental disorder as well as girls should receive special consideration. When asking for the symptoms of psychological burden, it is of utmost importance not only to ask the parents but also the children and adolescents themselves. Telepsychiatry and support of the continuity of care in schools and institutions might represent promising and necessary measures to cope with the upcoming challenges during pandemic crises, particularly in children and adolescents with mental disorders.

## Data Availability Statement

The raw data supporting the conclusions of this article will be made available by the authors, without undue reservation.

## Ethics Statement

Parental informed consent and children's assent were obtained, and the study was approved by the local Ethical Committee (EK 218/20) and conducted in accordance with the Code of Ethics of the World Medical Association (Declaration of Helsinki). Written informed consent to participate in this study was provided by the participants' legal guardian/next of kin.

## Author Contributions

BHD, KK, and SG: conceived and designed the experiments. SG and KK: wrote the paper and analyzed the data. SG: recruitment and data collection. All authors contributed to the article and approved the submitted version.

## Conflict of Interest

The authors declare that the research was conducted in the absence of any commercial or financial relationships that could be construed as a potential conflict of interest.

## Publisher's Note

All claims expressed in this article are solely those of the authors and do not necessarily represent those of their affiliated organizations, or those of the publisher, the editors and the reviewers. Any product that may be evaluated in this article, or claim that may be made by its manufacturer, is not guaranteed or endorsed by the publisher.
